# Impact of surgeon handedness in manual and robot-assisted total hip arthroplasty

**DOI:** 10.1186/s13018-020-01671-0

**Published:** 2020-04-21

**Authors:** Xiangpeng Kong, Minzhi Yang, Xiang Li, Ming Ni, Guoqiang Zhang, Jiying Chen, Wei Chai

**Affiliations:** 1 Department of Orthopaedics, Chinese PLA General Hospital, No. 28 Fuxing Road, Haidian, Beijing, 100853 China; 2 Medical College, Nankai University, Tianjin, China

**Keywords:** Total hip arthroplasty, Robotic-assisted surgery, Handedness, Acetabular component position

## Abstract

**Background:**

The purpose of this study was to examine whether surgeon handedness could affect cup positioning in manual total hip arthroplasty (THA), and whether robot could diminish or eliminate the impact of surgeon handedness on cup positioning in robot-assisted THA.

**Methods:**

Fifty-three patients who underwent bilateral robot-assisted THA and sixty-two patients who underwent bilateral manual THA between August 2018 and July 2019 in our institute were respectively analyzed in this study. When the difference between the bilateral anteversion and inclination was greater than 5°, the patient was regarded as having different cup positioning between bilateral THA. Their demographics, orientation of acetabular cup, and postoperative 3 month Harris hip score (HHS) were recorded for analysis.

**Results:**

There were no significant differences in the gender, age, BMI, diagnosis’s composition, and preoperative and postoperative HHS between the robotic and manual group. Two left hips dislocated in the manual group. The anteversion of left hip was significantly larger than that of right hip (24.77 ± 10.44 vs 22.44 ± 8.67, *p* = 0.043) in the manual group. There were no significant differences of cup positioning between bilateral robot-assisted THA. The patients in manual group were significantly more likely to have different cup positioning between bilateral hips than those in robotic group (77% vs 45%, *p* = 0.000). More manual THA were located out of the target zone than robot-assisted THA (70% vs 48%, *p* = 0.001).

**Conclusions:**

Surgeon’s handedness showed a trend towards an impact on cup positioning in manual THA and robot might help surgeon eliminate the adverse impact. However, the impact of handedness on the clinical outcomes still needs further observation.

## Background

Handedness, the tendency to use one hand more skillfully or in preference to the other, is one unique psychomotor manifestation. The obvious influence of handedness on surgical procedures and clinical outcomes in general surgery, dentistry, and urology has been reported in multiple literatures [1–5].

The human’s bones are symmetrically distributed, so the potential impact of surgeon’ handedness on orthopedic surgery may be even greater than non-orthopedic surgery [6]. Several studies showed that the right-handed surgeon was out of kilter when performing the left joint replacement [7–9].

In recent years, the semi-active haptic robotic systems, which could provide intraoperative tactile feedback to the surgeons, have earned widespread acceptance and significant growth in orthopedics [10]. Compared to trauma and spine, robot has entered a relatively mature stage in joint field and proved to have significant advantages on improving the accuracy of component placement, regardless of total hip arthroplasty (THA), total knee arthroplasty (TKA), or unicompartmental knee arthroplasty (UKA) [11, 12].

Although robot itself has no handedness, it requires the surgeon to register and manipulate the robotic arm to execute the surgical plan. To date, whether the surgeon’s handedness would influence the radiographic and clinical outcomes in robot-assisted THA is still unknown. In this study, the questions we sought to answer were as follows: (1) Could surgeon’s handedness affect cup positioning in manual THA? (2) Could robot eliminate the impact of surgeons’ handedness on cup positioning in robot-assisted THA?

## Patients and methods

In this study, we retrospectively reviewed the consecutive simultaneous bilateral manual THA and simultaneous bilateral robot-assisted THA in our institute between August 2018 and July 2019. The right hips were operated firstly in all the patients. Inclusion criteria : (1) all surgeries were performed through the posterolateral approach, (2) bilateral THA were completed by the same surgeon with the same prosthesis, and (3) bilateral hips had the same stage of the same etiology (Crowe classification and Ficat classification) and bilateral acetabulum had similar bone mass [13, 14]. Exclusion criteria: (1) previous surgery or fracture of either hip, (2) the target angles of bilateral acetabular cups were different, (3) the patients with ankylosing spondylitis, and (4) the patients with incomplete clinical data or nonstandard radiographs. All acetabular cups were aimed to place at 20° (anteversion) and 40° (inclination). The surgeon might adjust slightly the position of the acetabular cup according to the intraoperative situation. If the final planned or target angle of bilateral sides were different, the patient would be excluded from this study. Mako robot (Stryker, Mahwah, USA) was adopted in this study. Institutional review board approval was obtained prior to initiation of this study.

A total of two senior surgeons were enrolled in this study (CJY and CW) and both of them were experienced surgeons in joint replacement (the total surgical volume of primary manual THA was greater than 1000, respectively).

Both of the surgeons were right-handed (Edinburgh Handedness Inventory) and always stood behind the patients when performed THA [15].

### Surgical procedure (robot-assisted THA)

The surgical procedure was described in one previous study [16]. Three reference pins were inserted into the iliac crest for attachment of the fixed pelvic array and a fixed adhesive electrode attached to the patellar of the operated leg for intraoperative assessment. All surgeries were performed through posterolateral approach under general anesthesia. The surgeon began the skin incision and preliminary exposure after attaching the pelvic arrays. Prior to hip dislocation, the proximal and dismal femoral checkpoint were captured to measure the preoperative leg length and hip offset. The surgeon then dislocated the joint and performed the femoral neck osteotomy. The position of the pelvis was confirmed by registering and verifying the position of patient-specific anatomical landmarks displayed on screen. The accuracy of the registration was confirmed using the validation spheres. A surgeon-controlled robotic arm was used to guide cup positioning. Finally, acetabular screws and the liner were impacted in place. The femur was prepared manually. Hip stability was tested through the full range of movement. Leg length and offset were checked clinically before implantation of final femoral stem and femoral head. The contralateral THA was performed according to the same surgical plan.

### Surgical procedure (manual THA)

The procedures of exposure and osteotomy were described above. The smallest reamer was used to determine the acetabular bottom, then the larger reamers in turn to prepare the acetabulum. The acetabular cup and femoral stem were implanted manually. Hip stability and leg length were tested through the full range of movement. Then the surgeon performed the contralateral THA as the first side.

The patients were followed at 3 months after surgery and took the x-rays of anteroposterior pelvic in supine position. The demographics, radiographic, and surgical data of each patient were collected, including gender, age, body mass index (BMI), diagnosis, orientation of acetabular cup, postoperative complications, and Harris hip score (HHS).

When taking postoperative x-rays, the hips were in 10 to 15° of internal rotation and the x-ray beam centered over the pubic symphysis. The longitudinal axis of the body and legs was parallel to the imaging table. The ceramic femoral head was used to calibrate the radiographs to eliminate magnification error. The following measurements were made.

Orientation of acetabular cup was measured with Orthoview Systems (Version 6.6.1, Materialise, Leuven, Belgium). The accuracy of this software for measuring inclination and anteversion has been reported [8, 17, 18].

Anteversion was the angle between the short and long axes of the ellipse projected by the cup. Anteversion = arcsin (short axis/long axis). Inclination of cup was the angle between the cup’s long axis and the trans-teardrop line or the trans-ischial tuberosity line [19, 20]. Cup malposition was defined when anteversion or inclination beyond the target zone (anteversion 15–25°; inclination 35–45°). When the difference between the bilateral anteversion or inclination was greater than 5°, the patient was regarded as having different cup positioning in bilateral hips.

The postoperative complications were defined as cup malposition, dislocation, aseptic loosening, periprosthetic joint infection (PJI), and re-operation.

All of the measurements were initially performed in a random order independently by two trained joint surgery residents (KXP and YMZ), who then made the measurements again after 2 weeks. The average of four values was regarded as the final result. When the difference between the average of the single angle measured by two residents was greater than 5°, the two residents measured together as the final value.

All statistical analyses were performed by SPSS version 22 (Inc., Chicago, IL, USA). Measurement data are shown as the mean, standard deviation, and extreme value. Measurement data were analyzed by paired student’s tests or rank sum test. Count data were analyzed by Fisher’s exact test and chi-square test. The agreement of intraobserver and interobservers was calculated by interclass correlation coefficient (ICC). A *p* value < 0.05 was considered significant for all analyses.

## Results

Sixty-two patients who underwent bilateral manual THA and fifty-three patients who underwent bilateral robot-assisted THA were enrolled in this study (Table 1). There were no significant differences in the gender, age, BMI, diagnosis’s composition, and preoperative and postoperative HHS between robotic group and manual group. One hip in the manual group occurred acute PJI, and was treated with debridement and irrigation. Two left hips in the manual group occurred dislocation and wore anti-rotation shoes for 2 months after manual reduction. There was no infection or dislocation in the robotic group.

**Table 1:** The basic information of patients in this study

Demography	Robotic group	Manual group	*p*
M:F	31:22	38:24	0.760
Age, years (SD, range)	42.91 (11.22, 29–77)	40.18 (11.46, 21–67)	0.201
BMI, kg/m^2^ (SD, range)	22.54 (2.88, 17.02–28.34)	21.91 (2.89, 17.93–29.21)	0.241
Preoperative HHS	39.99 (12.06, 21–61)	41.62 (13.64, 23–63)	0.794
Diagnosis			
ONFH (%)	30/53 (56.60%)	42/62 (67.74%)	0.280
DDH (%)	21/53 (39.62%)	16/62 (25.81%)	
Others (%)	2/53 (3.77%)	4/62 (6.45%)	
Postoperative HHS	84.14 (6.29, 72–96)	82.37 (9.11, 68–95)	0.231

*SD* standard deviation, *ONFH* osteonecrosis of the femoral head, *DDH* developmental dysplasia of hip

**Fig. 1:**
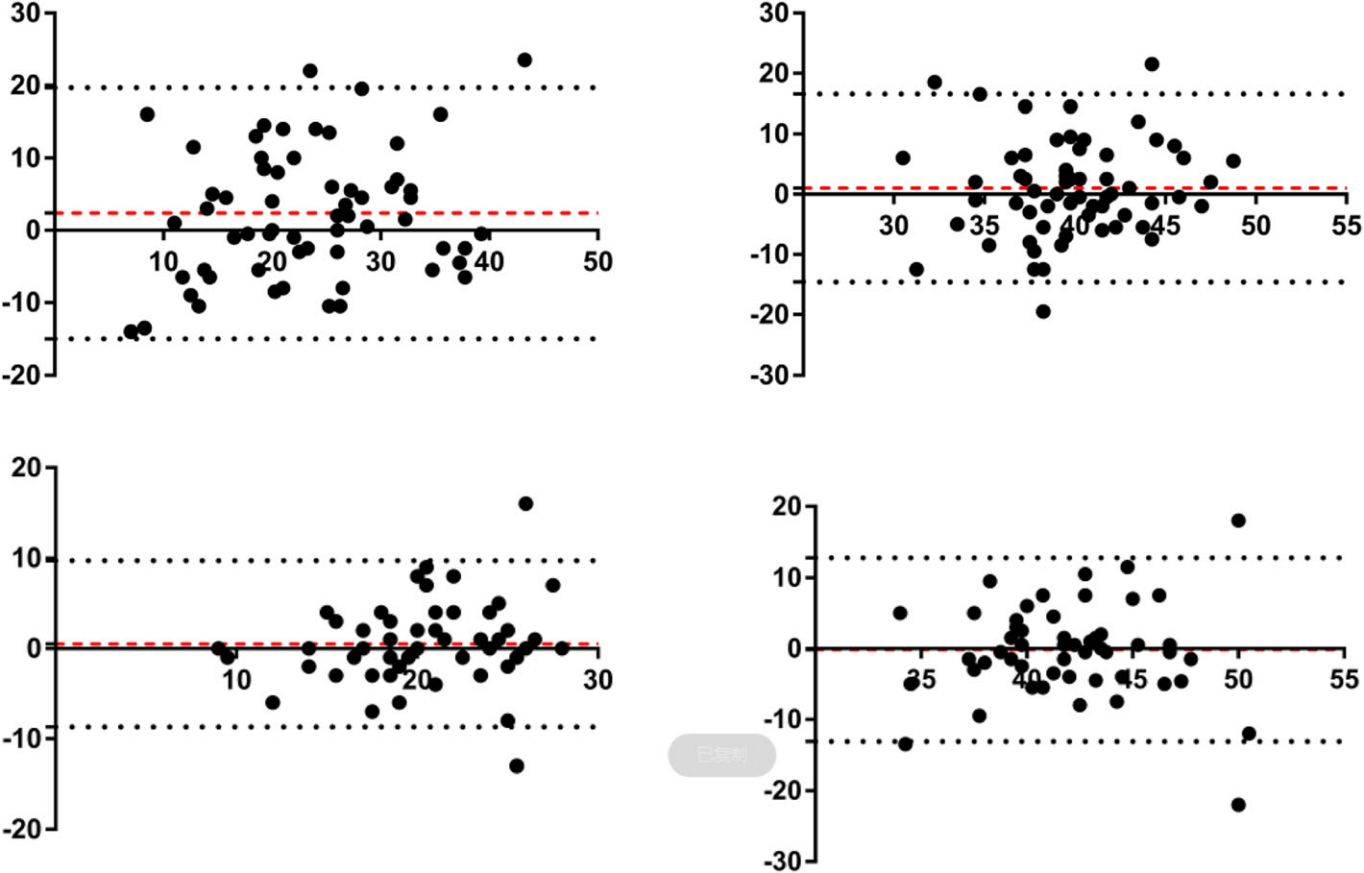
The Bland-Altman plot of bilateral cup positioning in manual and robot-assisted THA (difference between bilateral cup positioning: (left minus right) anteversion or inclination).Upper left, the anteversion in manual group; upper right, the inclination in robotic group; lower left, the anteversion in robotic group; lower right, the inclination in robotic group

In manual group, the anteversion of left hip was significantly larger than right hip (24.77 ± 10.44 vs 22.44 ± 8.67, *p* = 0.043). There were no significant differences of inclination, cups locating out of target zone, or HHS between bilateral hips. The left cups were more likely to be placed in larger anteversion by the right-handed surgeons (Table 2). The bilateral anteversion in manual group had poor consistency and were positive-skewed distributed (Fig. 1).

**Table 2:** Comparison of cup positioning in bilateral manual THA

Group	Left hip	Right hip	*p*
Anteversion (°)	24.77 (10.44, 0–55)	22.44 (8.67, 1–41)	0.043
Inclination (°)	40.35 (5.77, 25–55)	39.35 (5.26,23–48)	0.321
Out of target zone	45/62	42/62	0.556
Postoperative HHS	81.11 (9.30, 68–95)	83.63 (9.02, 71–95)	0.166

In robotic group, the bilateral cup positioning in robotic group had higher consistency (Fig. 1). There were no significant differences of cup positioning and HHS between bilateral hips, whether the anteversion, inclination, or cups locating out of target zone. With the assist of robot, the cup positioning was not influenced by the surgeons’ handedness (Table 3).

**Table 3:** Comparison of cup positioning in bilateral robot-assisted THA

Group	Left hip	Right hip	*p*
Anteversion (°)	20.72 (5.26, 9–34)	20.19 (4.66, 9–32)	0.417
Inclination (°)	41.90 (4.98, 28–59)	42.04 (5.13, 32–61)	0.875
Out of target zone	27/53	24/53	0.560
Postoperative HHS	83.44 (6.21, 72–94)	84.84 (6.38, 73–96)	0.332

**Fig. 2:**
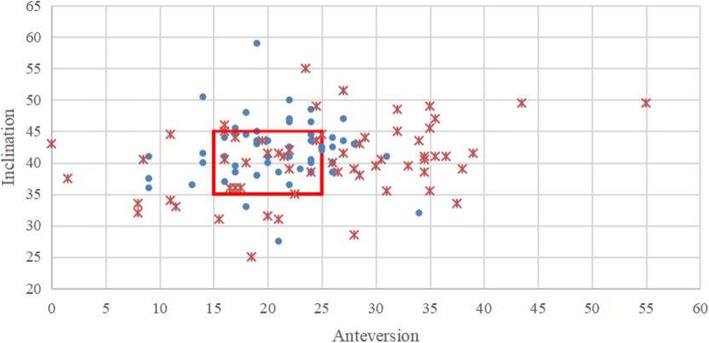
The scatterplot of inclination and anteversion in left robot-assisted and left manual THA. (The red box represented the target zone; **.** represented robot-assisted THA; *represented manual THA)

**Fig. 3:**
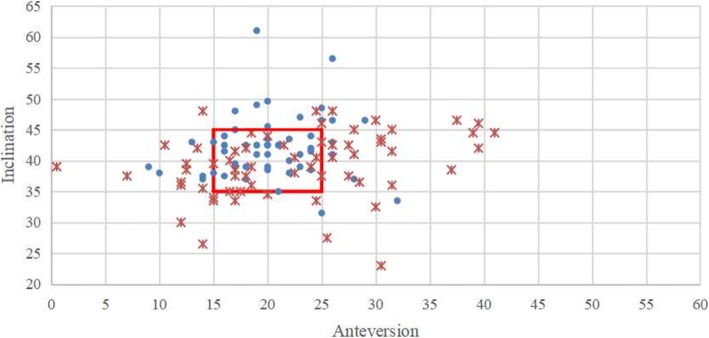
The scatterplot of inclination and anteversion in right robot-assisted and right manual THA. (The red box represented the target zone; **.** represented robot-assisted THA; *represented manual THA)

The patients undergoing bilateral manual THA were significantly more likely to have different cup positioning between bilateral hips than the patients undergoing bilateral robot-assisted THA (77% vs 45%, *p* = 0.000). Furthermore, More manual THA were located out of the target zone than robot-assisted THA (70% vs 48%, *p* = 0.001), whether left (73% vs 51%, *p* = 0.017) or right (68% vs 45%, *p* = 0.015). Robot-assisted THA was more stable in cup positioning than manual THA (Table 4, Figs. 2 and 3).

**Table 4:** Comparison of the difference between bilateral cup positioning in robot-assisted and manual THA

Group	Robotic group	Manual group	*p*
Difference of bilateral anteversion (°)	0.53 (4.71, − 13–16)	2.34 (8.91, − 17–24)	0.168
Difference of bilateral inclination (°)	− 0.14 (6.62, − 22–18)	1.01 (7.94, − 20–22)	0.405
Difference of bilateral positioning > 5°	24/53	48/62	0.000
Out of target zone	51/106	87/124	0.001

Difference between bilateral cup positioning: (left minus right) anteversion or inclination

The intraobserver and interobserver agreements were found to have nearly perfect reliability for all of the measurements (ICC > 0.81). The results are shown in Table 5.

**Table 5:** Intraobserver and interobserver variations of measurements

Observer	Inclination (95% CI)	Anteversion (95% CI)
KXP	0.94(0.92–0.96)	0.88(0.82–0.92)
YMZ	0.90(0.86–0.93)	0.85(0.78–0.89)
Interobserver	0.92(0.88–0.94)	0.85(0.78–0.90)

*CI* confidence interval

## Discussion

Surgeons are used to performing hip replacements in the dominant side and their preference caused by handedness had further adverse impact on cup positioning in the non-dominant side. Right-handed surgeons were more likely to place the left cup in larger anteversion (24.77 ± 10.44 vs 22.44 ± 8.67, *p* = 0.043). The patients in manual group were significantly more likely to have different cup positioning between bilateral hips than those in robotic group (77% vs 45%, *p* = 0.000). More manual THA were located out of the target zone than robot-assisted THA (70% vs 48%, *p* = 0.001). Robot-assisted THA was more stable in cup positioning than manual THA and robot might help surgeon eliminate the adverse impact of personal innate handedness. The above results were the important findings of this study.

Handedness is also called the lateralization of chirality, which is defined as the property of using one hand more than the other [21]. The effect of handedness, which can be reflected in most of surgery, is not generally considered to have a significant effect on their surgical outcome, because surgeons can adjust their positioning and perspective to optimize intracorporeal maneuverability [21]. However, the impact of handedness on orthopedic surgery is far greater than other surgeries. Bones are symmetrical anatomical structures and different from the internal organs in a constant position of the body [6, 7]. The surgeon’s standing position during operation will directly affect the accuracy of spatial positioning and an unaccustomed perspective will further lead to visual errors. Furthermore, the division of labor between the right and left hands is radically distinct during the bilateral orthopedic surgeries. When the non-dominant hand dominates one surgical procedure, it could compromise the surgical performance and clinical outcomes.

In 1994, Moloney et al. first reported the impact of handedness on the surgical outcome in orthopedics [6]. They concluded that malpositioning of the failures occurred significantly more frequently on the left than on the right, in a unit where all the surgeons were right-handed.

In 2014, Pennington et al. first reported that surgeon handedness appeared to influence acetabular component position during THA after analyzing 160 patients who were operated by 4 surgeons [9]. However, their study had several obvious drawbacks. The sample size of single surgeon was relatively small. No demographic comparisons between the patients who underwent different sides of THA. The type and fixation of the prosthesis were also not controlled. There was only one observer and no repetitive measurement to perform consistency analysis. Furthermore, the outcome did not include the anterversion, functional score, and complications.

Current debates regarding optimal position of the acetabular cup remained unsolved. Several surgeons have put forward the safe zones for inclination and anteversion respectively [22, 23]. The most commonly used safe zone was established by Lewinnek et al. (anteversion 5–25°; inclination 30–50°) in 1978 [22]. However, recent studies have reported that an inclination of 45° or greater was associated with a significant increase in linear wear per year compared with an inclination less than 45° [24, 25]. Thus, Callanan et al. redefined the safe zone (anteversion 5–25°; inclination 30–45°) in 2011 [23].

In 2019, Crawford et al. compared the acetabular component position differences between right and left hips for a right-hand dominant surgeon [26]. In their study, right hips had a significantly lower abduction and less combined Lewinnek outliers through direct anterior approach, and right hips had significantly higher anteversion and Lewinnek anteversion outliers through posterolateral approach. Significant superiority of cup positioning was found in both approaches based on the surgeons’ dominant and non-dominant side. However, they also ignored an important influence factor, which was the comparability between the two sides of THA has not been fully established.

In this study, the target zone referred the above literature and was further reduced (anteversion 15–25°; inclination 35–45°). If the acetabular cup in one side was accurate (anteversion 20°; inclination 40°), the other side would reach the outlier with the difference of 5°. An inclination of 45° or greater would increase stress concentrations to degrade component durability [24, 25]. And that the robotic system’s acceptable error for cup anteversion and inclination was 5°. That’s why we used a difference of 5° as the cutoff to define the different cup positioning. We enrolled the patients who underwent the simultaneous bilateral THA to avoid the difference of the acetabular bone mass and demographic differences between the patients who underwent unilateral THA. Another strength of this study was that we included the anteversion, functional score, and complications. Finally, we compared the robot-assisted with manual THA to verify the robot’s advantage in eliminating handedness.

In manual THA, cup positioning comes from first the angular proprioception and then manual implantation. The previous study in our institute showed that the placement of cup performed by dominant hands is more accurate than that performed by non-dominant sides [8].This study also confirmed this result. The surgeons’ handedness had significant influence on cup positioning and right-handed surgeons were more likely to place the left cup in larger anteversion in manual THA. As Kanawade et al. reported, they performed robot-assisted THA in 38 patients (43 hips) and measured the cup positioning by postoperative CT scans. There were 12% and 16% outlier of 5° in inclination and anteversion respectively [28]. However, whether the more accurate cup positioning by robot could improve the patients’ clinical outcomes remained to be seen. In this study, no significant difference of postoperative HHS between bilateral THA was found. Even so, we should be aware of the potential side-effect that may be introduced by the surgeon’s handedness and laterality of operated extremities. Each surgeon should consider taking extra precautions to diminish or eliminate the adverse results when operating on the non-dominant side [7]. Because the scoring system of Harris has two inherent disadvantages, namely ceiling effect and low sensitivity, the postoperative hip function and dislocation rate between bilateral cups may be significant with the refinement of scoring system, enlarging of sample size, and extension of follow-up period.

In the recent years, sophisticated tools have been emerging to reduce the differences between surgeons and sides. In addition to these tools, robot might be one alternative to eliminate the adverse influence of handedness [4, 21, 27]. However, as the surgeon has to use the non-dominant hand to complete critical procedures, such as acetabular registration, reaming, and cup implantation during robot-assisted THA of the non-dominant side, the surgeon’s handedness could still influence the cup positioning theoretically.

The results of this study found that the surgeons’ handedness had no significant influence on robotic cup positioning. While the bilateral cup positioning existed some deviation in robot-assisted THA, it had no inclination to either side. Robot was capable of eliminating the innate handedness in early practice of robot-assisted THA, regardless of the surgeon’s experience.

Intraoperative feedback mechanisms were the important factors which contributed to the consistent clinical outcome between bilateral robot-assisted THA. The robotic real-time feedback mechanism had powerful ability to help surgeons get rid of the limitations of visual spatial positioning.

In the future, medical training may be one of the promising directions of robot application in orthopedics [7, 29]. The undifferentiated performance of robot among different surgeons and different sides demonstrates its great potential role in the surgical training and education. In joint replacement, the perception of component positioning requires a lot of practice and immediate feedback to reach a steady state, especially on the non-dominant side. The adoption of robot could allow novice surgeons to form the correct sense of spatial orientation and reduce the risk of prosthetic malposition. The accumulation of experience and the progress of learning can be accelerated with the haptic feedback of semi-active robot.

This study has several limitations. Firstly, the measuring bias could not be ignored. Although the application of Orthoview systems in measuring anteversion and inclination was reported in several studies, the postoperative measurements basing on the x-rays were inferior than the computerized tomography (CT) and magnetic resonance imaging (MRI). Postoperative CT scan would have enabled more accurate assessment of radiographic outcomes. Secondly, both of the surgeons enrolled in this retrospective study were right-handed and had rich experience in joint replacement. The prospective studies including the left-handed and young surgeons should be conducted in future. Thirdly, the small sample size and short follow-up period might mask the possible differences. The significant difference of cup positioning did not bring out the significant change of clinical outcomes. Although the power of comparison of different cup positioning (0.96) and target zone ratio (0.93) was convincing, the power of comparison of anteversion was relative low (0.53), which affected the persuasion of the conclusion to some extent. Fourthly, dislocation is the result of various factors and combined femoral-acetabular components’ position is crucial important. In this study, the different proportions of DDH cases with presumably higher femoral anteversion between two groups may have influenced the dislocation rate.

## Conclusion

Surgeon’s handedness showed a trend towards an impact on cup positioning in manual THA and robot might help surgeon eliminate the adverse impact. However, the impact of handedness on the clinical outcomes still needs further observation.

## Data Availability

All data generated or analyzed during this study are included in this published article.

## Abbreviations

THA: Total hip arthroplasty
HHS: Harris hip score
TKA: Total knee arthroplasty
UKA: Unicompartmental knee arthroplasty
BMI: Body mass index
PJI: Periprosthetic joint infection
ICC: Interclass correlation coefficient
SD: Standard deviation
ONFH: Osteonecrosis of the femoral head
DDH: Developmental dysplasia of hip
CI: Confidence interval
CT: Computerized tomography
MRI: Magnetic resonance imaging
